# Folic Acid Functionalized Diallyl Trisulfide–Solid Lipid Nanoparticles for Targeting Triple Negative Breast Cancer

**DOI:** 10.3390/molecules28031393

**Published:** 2023-02-01

**Authors:** Anindita De, Parikshit Roychowdhury, Nihar Ranjan Bhuyan, Young Tag Ko, Sachin Kumar Singh, Kamal Dua, Gowthamarajan Kuppusamy

**Affiliations:** 1 College of Pharmacy, Gachon Institute of Pharmaceutical Science, Gachon University, Incheon 21936, Republic of Korea; 2 Department of Pharmaceutical Chemistry, Himalayan Pharmacy Institute, Majitar 737136, East Sikkim, India; 3 School of Pharmaceutical Sciences, Lovely Professional University, Phagwara 144411, Punjab, India; 4 Faculty of Health, Australian Research Centre in Complementary & Integrative Medicine, University of Technology Sydney, Ultimo, NSW 2007, Australia; 5 Discipline of Pharmacy, Graduate School of Health, University of Technology Sydney, Ultimo, NSW 2007, Australia; 6 Department of Pharmaceutics, JSS College of Pharmacy, JSS Academy of Higher Education & Research, Ooty 643001, Tamil Nadu, India

**Keywords:** diallyl tri-sulfide, folic acid, solid lipid nanoparticles, TNBC, cellular internalization and cell migration, Bcl2 apoptosis protein

## Abstract

DATS (diallyl trisulfide), an anti-oxidant and cytotoxic chemical derived from the plant garlic, has been found to have potential therapeutic activity against triple-negative breast cancer (TNBC). Its hydrophobicity, short half-life, lack of target selectivity, and limited bioavailability at the tumor site limit its efficacy in treating TNBC. Overexpression of the Folate receptor on the surface of TNBC is a well-known target receptor for overcoming off-targeting, and lipid nanoparticles solve the limitations of limited bioavailability and short half-life. In order to overcome these constraints, we developed folic acid (FA)-conjugated DATS-SLNs in this research. The design of experiment (DoE) method was employed to optimize the FA-DATS-SLNs’ nanoformulation, which resulted in a particle size of 168.2 ± 3.78 nm and a DATS entrapment of 71.91 ± 6.27%. The similarity index between MCF-7 and MDA-MB-231 cell lines demonstrates that FA-DATS-SLNs are more therapeutically efficacious in the treatment of aggravating TNBC. Higher cellular internalization and efficient Bcl2 protein downregulation support the hypothesis that functionalization of the FA on the surface of DATS-SLNs improves anticancer efficacy when compared with DATS and DATS-SLNs. FA-functionalized DATS-SLNs have demonstrated to be a promising therapeutic strategy for TNBC management.

## 1. Introduction

Breast cancer (BC) is one of the most common invasive tumors among women globally and is one of the prime causes of malignancy-related death. Men account for less than 1% of all diagnosed BC cases [1,2,3]. As per the latest GLOBOCAN data, BC will rise by 41% in incidence and by 59% in mortality if remained unchecked in the next 20 years. Currently, the highest mortality is seen in developing countries such as Barbados and Fiji whereas, in incidence, European countries such as Belgium and the Netherlands top the chart. BC is an etiologically and clinically complex disorder. With the development of screening mammography and effective therapy over the last few decades, the rate of survival has improved. However, overall success is still far away due to the heterogenic nature of the BC, drug resistance, and lack of novel targeted biomarkers. 

The term “triple-negative breast cancer” (TNBC) refers to a kind of BC in which the Human Epidermal Growth Factor Receptor 2 (HER-2), Progesterone Receptor (PR), and Estrogen Receptor (ER) are not expressed in the tumors [4]. Among the several BC subtypes, TNBC is significant for being the most aggressive and invasive. For people with TNBC, there is currently no clinical treatment available. Platinum agents, taxanes, anthracyclines, new microtubule stabilizing agents, and other DNA-damaging regimens are some of the current TNBC therapeutic options [5]. However, intrinsic or acquired resistance, as well as cross-resistance to other therapeutic medications, is expected to complicate these approaches. As a result, ongoing research aimed at finding innovative possible treatment pathways for enhancing outcomes in TNBC promises to be very advantageous. Finding medicinal and edible plant resources that target the indicators and detect biological markers of TNBC progression may thus be significant for the prevention of BC metastasis, enabling alternative therapeutic approaches for the condition [6]. Currently, to minimize the toxicity profile of synthetic compounds, researchers are concentrating on dietary phytochemicals as a therapeutic strategy for cancer cell targeting. Organosulfur compounds (OSCs) derived from Allium plants, including garlic (*Allium sativum*) and onion (*Allium cepa*), have been widely studied for their cytotoxic effects against various forms of cancer. The cellular mechanism for anticancer activity is dependent [5,7,8] on bio-activating processes that prevent the carcinogen from covalently attaching to cellular DNA [9]. Among the various phytochemicals investigated, Diallyl Trisulfide (DATS), a high organosulfur component found in garlic, has lately caught researchers’ interest [5,8]. Research has previously demonstrated that DATS is an effective cytotoxic agent. This plant-derived chemical, on the other hand, is poorly water soluble, off-target, has a low bioavailability, a short biological half-life, and poor stability. To address this limitation, a target-specific, stable formulation with high DATS loading and low toxicity is required. 

Solid lipid nanoparticles (SLNs) [10,11] are gaining popularity among a wide variety of lipid particles due to their distinct safe lipid profiles, high drug loading, and better protection of the incorporated phyto-compounds from adverse biological conditions. The solid core releases therapeutics in a very controlled manner over a long period. 

Research has previously demonstrated the therapeutic effectiveness of DATS in treating BC, but it has also raised questions concerning target selectivity and tumor accumulation efficiency. Folate receptor (FR) is a glycosylphosphatidylinositol-linked membrane glycoprotein that is overexpressed in BC and acts as a biomarker [12]. Folic acid (FA) enters the cytoplasm by the FR-mediated endocytosis process [13]. The process of FA conjugation is widely understood and extensively demonstrated [14]. In this study, we used 1,1’-carbonyldiimidazole (CDI) to chemically conjugate FA with the surfactant, which was used to form the surface shell. Because the surfactant forms the outer layer of the DATS-SLNs, it was first functionalized with FA. The FTIR and NMR analyses validated the functionalization. The optimization of the formulation was conducted using the Design of Experiment (DoE) approach. To evaluate the efficacy of functionalization for TNBC targeting, DATS-SLNs and FA-DATS-SLNs were compared in cancer cell lines. We can speculate that FA-functionalized DATS-loaded SLNs could be a promising candidate for targeting and specifically delivering cytotoxic drugs to aggressive TNBC. This research article focused on the development of FA-functionalized DATS-loaded SLNs to target TNBC cell apoptosis and high cellular internalization with high DATS encapsulation and prolonged circulation time without being toxic to healthy cells.

## 2. Results

### 2.1. Lipids Selection for FA-DATS-SLNs Formulation

**Figure 1:**
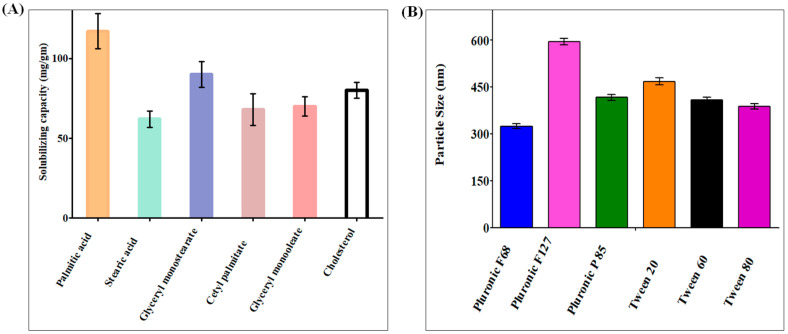
(**A**) Solubilizing capacity of DATS in palmitic acid, stearic acid, glyceryl monostearate, glyceryl monooleate, acetyl palmitate, and cholesterol. The data show that DATS had the highest solubilizing capacity in palmitic acid. (**B**) DATS SLNs was developed using Pluronic F-68, Pluronic F-127, Pluronic P-85, Tween 20, Tween 60, and Tween 80. The graphical data indicate that Pluronic F68 was the most suitable surfactant to produce minimum PS.

The capacity of SLNs to encapsulate a certain drug is significantly influenced by the drug’s solubilizing capacity within the solid lipid core, as the larger the solubilizing capacity, the greater the drug loading potential. A total of six solid lipids were used in the solubilizing capacity investigation, with palmitic acid having the highest solubilizing capacity compared with the other lipids (Figure 1A). Palmitic acid had the highest drug solubilizing capability of 117.5 ± 10 mg/gm of lipid, whereas cetyl palmitate had the second highest at 68.3 ± 5 mg/gm of lipid. Triglycerides have numerous benefits as the backbone of lipid-based delivery systems. They are regularly taken in food, are totally digested and absorbed, and hence pose no health risks [15]. Palmitic acid has also been reported to be utilized in the manufacture of SLNs throughout recent research [16].

### 2.2. Surfactant Selection for FA-DATS-SLNs Formulation

SLNs are colloidal nanoparticle systems that are stabilized in aqueous environments by surfactants and have a matrix medium composed of solid lipids. Surfactants in the SLNs promote endothelial cell permeability by promoting lipid solubilization and membrane fluidization. Nanoparticles were formulated with six different surfactants and analyzed for particle size (PS) and polydispersity index (PDI), using palmitic acid as the solid lipid for surfactant selection. Pluronic F68 has the smallest PS and PDI, as shown in Figure 1B. Poloxamers are nonionic triblock copolymers made up of a hydrophobic polyoxypropylene chain in the center and two hydrophilic polyoxyethylene chains on either side. Furthermore, poloxamers have been shown to sterically stabilize nanoparticles, inhibit plasma proteins or opsonins from attaching to nanoparticle surfaces, and provide nanoparticles with a hydrophilic characteristic, which can prevent medications containing SLNs from leaving circulation. When compared with single-chain and ionic surfactants, large and nonionic surfactants generally cause less toxicity [17,18]. Because of our previous research on SLNs, we selected soy lecithin as a co-surfactant for this study.

### 2.3. DATS- Lipid-Surfactant Compatibility Analysis

The compatibility of all of the selected ingredients is critical for maximal therapeutic success. The IR spectra (Figure S1) of DATS exhibited absorption bands at 3086, 2976, and 719 cm^−1^, corresponded to the -CH stretching and bending. Palmitic acid’s characteristic peaks were -CH stretch at 2915 cm^−1^, -OH stretch at 2662 cm^−1^, C=O stretch at 1689 cm^−1^, and -OH stretch at 937 cm^−1^. There were no significant differences in the peak characteristics of DATS and palmitic acid in DATS-SLNs formulated with palmitic acid, confirming that solid lipids and DATS are extremely compatible in formulation conditions. 

### 2.4. DoE Approach for the Optimization of DATS-SLNs

**Figure 2:**
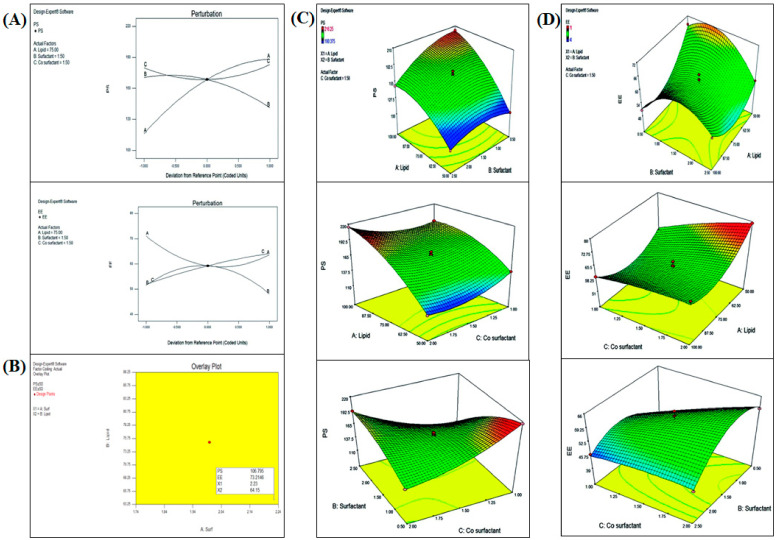
(**A**) Perturbation plot depicting the effects of solid lipid, surfactant, and co-surfactant on PS and %EE. (**B**) Overlay plot of the BBD model for the DSTA-SLNs. (**C**) Response surface plots of PS, indicating the effects of lipid, surfactant, and co-surfactant. (**D**) Response surface plots of %EE, indicating the effects of lipid, surfactant, and co-surfactant.

There was a substantial shift in PS and % entrapment efficiency (%EE) when the amounts of palmitic acid, Pluronic F68, and soy lecithin were changed. As a consequence, amounts of palmitic acid, Pluronic F68 concentration, and soy lecithin concentration were selected as independent variables for Box–Behnken Design (BBD) optimization of DATS-SLNs. Using the BBD experimental methodology, we optimized the DATS-SLNs formulation by varying the lipid, surfactant, and co-surfactant ratios. The responses recorded for each of the 17 formulations were evaluated for optimization (Table S1). Using perturbation plots and response surface plots, the effects of independent variables on PS and EE were investigated (Figure 2). An analysis of variance (ANOVA) was used to validate the BBD model (Tables S2 and S3). The optimal formulation was chosen based on the PS (nm) and %EE desirability criteria.

The overlay plot of the BBD model of the DATS-SLNs actually demonstrates that 64.15 mg of palmitic acid lipid and 2.23% surfactant along with 0.4% soy lecithin were capable of producing a minimum PS of 108.12 ± 5.45 nm (Figure S2) (predicted data 106.79 nm) with a %EE of 72.67 ± 4.61% (predicted data 73.21%). The observed findings are in close accord with the design’s BBD-model projected values.

### 2.5. Functionalization of Folic Acid to Formulate FA-DATS-SLNs

To increase their target specificity for TNBC, the DATS-SLNs were functionalized with FA to target overexpressed FR. The outer layer that covers the drug-loaded solid lipid core is created by the surfactant and co-surfactant; thus, the surfactant Pluronic F68 was functionalized with FA for target specificity [19].

**Figure 3:**
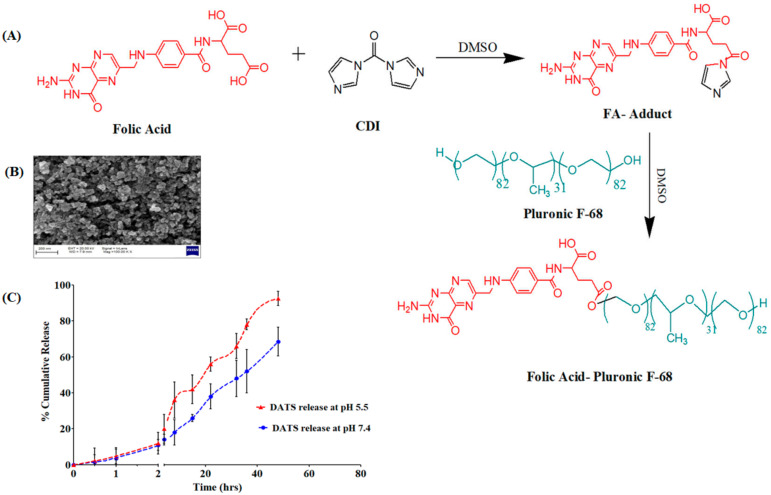
(**A**) Synthesis pathway of the FA-Pluronic F-68 surfactant for the formulation of DATS-SLNs. (**B**) SEM image of FA-DATS-SLNs for the morphology study. (**C**) *In vitro* DATS release in two different pH conditions.

The -COOH group of the FA is activated by CDI and forms an FA-CDI adduct with an activated carbonyldiimidazole group. The active adduct then reacts with the -OH group of the terminal PEG chain of Pluronic F-68. The molar ratio of FA:CDI:Pluronic F68 was chosen at 1:2:5 to confirm that at least one of the -OH groups of Pluronic F-68 must be conjugated to FA [20]. The conjugation’s reaction scheme is depicted in Figure 3A.

### 2.6. Characteristics Evacuation of FA-DATS-SLNs–FTIR, NMR, Entrapment Efficiency, Particle size and Morphology

#### 2.6.1. FTIR Conformation for Functionalization of FA on DATS-SLNs

The FTIR analysis verified the surface functionalization of the DATS-SLNs with FA (Figure S3A). For palmitic acid, the FTIR revealed characteristic peaks of -CH stretch at 2915 cm^−1^, -OH stretch at 2662 cm^−1^, and C=O stretch at 1689 cm^−1^. The FA has two characteristic bands that correspond to the -NH2 group at 3321 cm^−1^ and 3409 cm^−1^.The peak at 1690 cm^−1^ corresponds to the carboxylic acid group (-COOH). The FTIR spectra of FA-DATS-SLNs exhibited the characteristic peak of FA at 3409 cm^−1^ in comparison to DATS-SLNs, which indicates the functionalization of the FA on the surface of DATS-SLNs.

^1^H NMR validated the synthesis of FA-Pluronic F68. The ^1^H NMR spectra of Pluronic F68, FA, and FA-Pluronic F68 are shown in Figure S3B. The typical peaks of PPG in the ^1^H NMR spectra of Pluronic F68 (Figure S3B.a) range from 1.2 ppm to 3.4 ppm, including signal (δ = 1.21 ppm) represent -CH_3_ group, signal c (δ = 3.38 ppm) represent -CH_2_ group, and signal d (δ = 3.41 ppm) for the -CH group. The polyethylene oxide (PEG) signals (a and b) were the -CH groups with a chemical shift of around 3.4 ppm.

The ^1^H NMR spectra of FA was presented in Figure S3B.b. The pteridine proton peaks l (δ = 8.5 ppm), k (δ = 7.6 ppm), aromatic proton peak m (δ= 6.6 ppm), and reactive -COOH peak i (δ = 12.5 ppm) are clearly visible in the FA ^1^H NMR spectra. Figure S3B.c illustrates the ^1^H NMR spectra of FA-Pluronic F68, which exhibited all of the characteristic signals of Pluronic F68 as well as FA. The signal at I (δ = 12.5 ppm), which corresponds to the FA’s -COOH group, does not appear in the spectra of the FA-Pluronic F68 conjugate. The reason for the disappearance of the peak in FA-Pluronic F68 may be due to the chemical reaction between the –COOH of the FA and the –OH of the Pluronic F68. This clearly indicates that the FA was conjugated with the Pluronic F68, which functionalized the FA-DATS-SLNs.

#### 2.6.2. Entrapment Efficiency, Particle Size, Morphology and *In Vitro* Release Study of FA-DATS-SLNs

The FA conjugation has no influence on the encapsulation of the DATS in the solid core of the SLNs. The FA-DATS-SLNs had an entrapment of 71.91 ± 6.27%, which did not differ substantially from the DATS-SLNs. A difference in PS between the DATS-SLNs and the FA-DATS-SLNs is another evidence of functionalization. The PS of the FA-DATS-SLNs was 168.2 ± 3.78 nm (Figure S4), which might be attributable to surface functionalization. The increase in PS after FA functionalization might be due to the rearrangement of a shell lipid structure surrounding the core as well as FA conjugation on the surface. A PS with a diameter of less than 200 nm is desirable to penetrate the tumor membrane and gradually accumulate on the target site over time [21]. According to SEM investigations, FA-DATS-SLNs were monodispersed and spherical in form (Figure 3B). A little rough surface morphology was noticed.

The drug DATS was released from the FA-DATS-SLNs formulation in a controlled yet pH-dependent manner. Both pH 7.4 and pH 5.5 produced an initial burst release of 11% within 2 h. This burst release was caused by the presence of unencapsulated DATS on the SLNs’ surface. pH 5.5 mimics the simulated cancer settings, but pH 7.4 is considered a normal physiological pH. The pH-dependent ‘off-on’ switching of DATS release is triggered by repeated exposure to different release media at pH 7.4 and 5.5 [22]. In changing pH conditions, pH-sensitive FA-Pluronic F-68 regulates DATS release. Furthermore, fatty palmitic acid degrades quickly in an acidic environment compared with pH 7.4 due to protonation of the -COOH group [23]. DATS has a pH-sensitive impact as well. Because of the presence of two sulfide groups, DATS is alkaline in nature and more soluble at lower pH. As a response, the SLN’s encapsulated DATS has a high tendency to penetrate the lower pH release medium. A higher rate of DATS release in tumor cells would come from preferential release in an acidic environment. At pH 5.5, about 92.5 ± 4.23% DATS was released after 60 h, whereas only 48.2 ± 8.16% DATS was released at pH 7.4 (Figure 3C). This suggests that the acidic environment accelerated medication release, which is extremely favorable for TNBC drug delivery.

### 2.7. In Vitro Cell Line Study for Triple Negative Cancer Efficacy

#### 2.7.1. *In Vitro* Cytotoxicity Study for Evaluation of Functionalization

**Figure 4:**
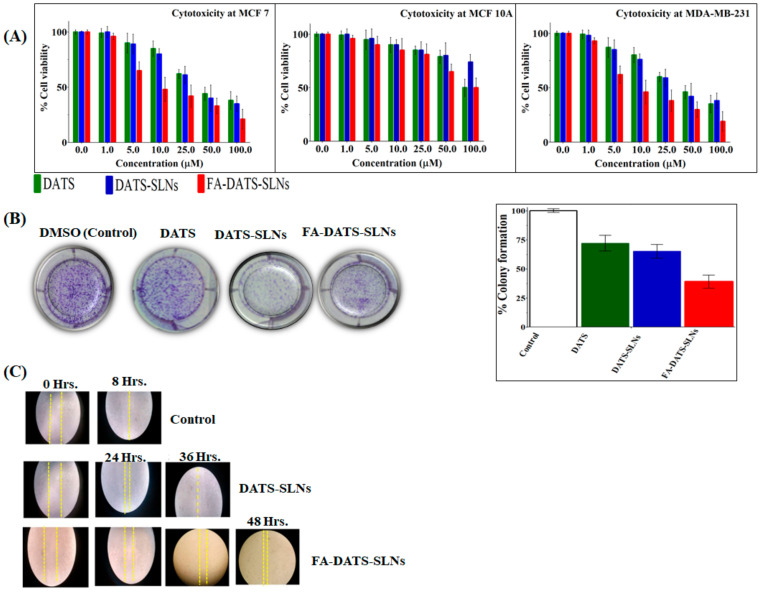
(**A**) Cytotoxicity studies in MCF-7, MCF-10A, and MDA-MB 231, and cell lines *in vitro* for 48 h. (**B**) MDA-MB-231 cell line colony formation assay. 1. Untreated cells (control); 2. cells treated with DATS; 3. cells treated with DATS-SLNs; 4. cells treated with FA-DATS-SLNs. The graphical representation indicates the % reduction of the colony formation compared with the control group (*p* < 0.01) after the treatment. (**C**) MDA-MB-231 cell migration assay for the formulation of DATS, DATS-SLNs, and FA-DATS-SLNs.

Different concentrations of DATS, DATS-SLNs, and FA-DATS-SLNs were studied for cancer cell growth (Figure 4A) for 48 h. Despite the fact that FR expression is a significant characteristic marker for both MCF-7 and MDA-MB-231 cells, aggressive TNBC is more challenging to target. The Selectivity Index (SI) in Table 1 shows that the cytotoxic effects of DATS, DATS-SLNs, and FA-DATS-SLNs selected for MDA-MB-231 cells indicated greater efficacies for the TNBC. The functionalized FA-DATS-SLNs formulation had a higher SI score of 13.2 for the MDA-MB-231 cell line compared with 8.7 for MCF-7 cancer cell lines.

The cytotoxicity of DATS-SLNs and FA-DATS-SLNs was dose-dependent. When compared with FA-DATS-SLNs, DATS-SLNs showed less cytotoxicity, which may be a result of the drug’s efflux into the cytoplasm through P-glycoprotein (P-gp) pumps. FA-DATS-SLNs might enter cells by receptor-mediated endocytosis and have no connection to P-gp efflux. As a result, the drug remains present inside the cells with a high level of cytotoxicity [24,25].

#### 2.7.2. Colony Formation Assay for Long-Term Cell Cytotoxicity Effects of Functionalization 

MDA-MB-231 cells treated with DATS, DATS-SLNs, and FA-DATS-SLNs demonstrated a decrease in colony formation compared with the control group (considered 100% colony formation) (Figure 4B), providing evidence of the long-term therapeutic efficacy of the functionalized formulation. MDA-MB-231 cells were treated for 24 h with DMSO (control), 7.1µg/mL concentration of DATS and the equivalent concentration of DATS in DATS-SLNs and FA-DATS-SLNs formulations, and then cultured in drug-free media for 7 days. Figure 4B illustrates the long term cytotoxicity efficacy for the MDA-MB-231 cells, with quantitative data presented graphically. FA-DATS-SLNs had fewer colonies than DATS-SLNs (*p* < 0.01), indicating that functionalized formulations may restrict proliferation, enhance DATS cellular internalization, and inhibit colony formation [26,27] better than DATS and DATS-SLNs over a prolonged period of time.

#### 2.7.3. Cancer Cell Migration Assay

The scratch distance of untreated MDA-MB-231 TNBC cells was lost in less than 8 h. The cell migration assay investigation of DATS-SLNs and FA-DATS-SLNs revealed that DATS-SLNs only prevented cell migration for 36 h, whereas FA functionalization on the surface of DATS-SLNs delayed cell migration until 48 h by promoting higher cellular internalization and more target specificity [28], demonstrating the advanced therapeutic application of the FA-DATS-SLNs over the non-functionalized formulation for TNBC management (Figure 4 C) (*p* ≤ 0.01 and *p* ≤ 0.001 under the Student *t*-test).

#### 2.7.4. DNA Fragmentation Analysis of Functionalization Efficacy for Apoptosis

**Figure 5:**
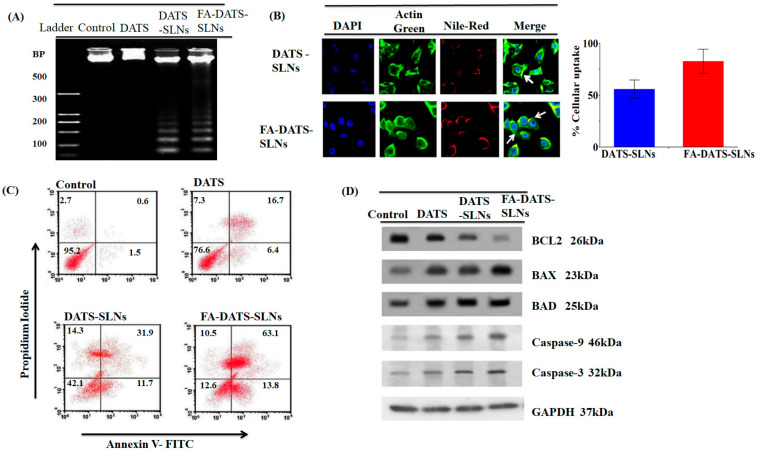
(**A**) DNA fragmentation of the MDA-MB-231 cell line using DATS, DATS-SLNs, and FA-DATS-SLNs. (**B**) Cellular internalization of DATS-SLNs and FA-DATS-SLNs in the MDA-MB-231 cancer cell line indicates that the functionalization of the SLNs surfaces enhances the cellular internalization in TNBC cells. (**C**) Quantitative measurement of apoptosis by AnnexinV-FITC versus PI in MDA-MB-231 cells treated with DATS, DATS-SLNs, and FA-DATS-SLNs. The data clearly indicate high late-phase apoptosis for the FA-DATS-SLNs compared with DATS-SLNs and DATS alone. (**D**) Bcl2-mediated apoptosis induced by FA-DATS-SLNs compared with DATS-SLNs and DATS.

After 24 h, the DNA was extracted, and the analysis was performed using 1% agarose gel electrophoresis (Figure 5A). The results clearly showed that MDA-MB-231 cell lines treated with DATS-SLNs and FA-DATS-SLNs exhibited more DNA fragmentation than the marker [21]. Faint fragmentation has also been observed in DATS therapy. The FA-DATS-SLNs may have a superior cellular accumulation of DATS since their concentration is higher due to the formulation being more target-specific. The DATS and the DATS-SLNs need to struggle with the P-gp efflux of the cells and lose therapeutic efficacy in a large manner. FA-DATS-SLNs enter cells by receptor-mediated endocytosis [3,29] and accumulate inside the cells in higher concentrations compared with DATS and DATS-SLNs. 

#### 2.7.5. TNBC Cellular Internalization of Functionalized FA-DATS-SLNs

The cellular absorption of DATS-SLNs and FA-DATS-SLNs was assessed in MDA-MB-231 TNBC cells using triple fluorescence labeling. This approach used three stains: the red fluorescence of Nile Red to mark SLNs, the green fluorescence of Actin-Tracker Green to label actin, and the blue fluorescence of DAPI to label the nucleus. Nile Red is a lipophilic dye that identifies the SLNs. Rows 1 and 2 illustrate the cellular uptake of DATS-SLNs and FA-DATS-SLNs, which were internalized in MDA-MB-231 TNBC cells (Figure 5B). When compared with the FA-DATS-SLNs in Rows 2, the DATS-SLNs had poor cellular internalization in Row 1. The mechanism for this was the functionalization of the SLNs’ surface with FA. The target specificity of the FA-DATS-SLNs boosted and improved DATS cellular internalization. FA tailored the formulation to the cancer cells, where the lipid SLNs formulation enhanced the penetration inside the cells. The quantitative cellular consumption of DATS-SLNs and FA-DATS-SLNs was therefore graphically represented and estimated from the fluorescence intensity of the CLSM data.

#### 2.7.6. Apoptosis Quantification of Functionalized FA-DATS-SLNs by Flow Cytometry

Annexin V-FITC/propidium iodide (PI) double labeling was used to quantify the SLNs’ apoptotic effect on the MDA-MB-231 cancer cell line (Figure 5C). PI, a fluorescent dye, only penetrated dead cells; it did not enter live cells. It is capable of accurately identifying cells in the apoptosis phase, including viable (Annexin V-and PI-, lower left square), early apoptotic (Annexin V+ and PI-, lower right square), late apoptotic (Annexin V+ and PI+, upper right square), and necrotic cells (Annexin V-and PI+, upper left square) [30]. Figure 5C shows that the use of DATS-SLNs and FA-DATS-SLNs enhanced the number of late apoptotic cells compared with DATS. The control group had 2.7% early and 0.6% late apoptotic cells, whereas the FA-DATS-SLNs group had 10.5% and 63.1%. DATS-SLNs had 14.3% and 31.9%, and only DATS had 7.3% and 16.7% of early and late apoptosis, respectively. The percentage of late apoptosis clearly indicates that FA functionalization enhances the apoptosis of the TNBC cell line. The FA-DATS-SLNs may provide higher localization and target specificity than DATS-SLNs and DATS. To enter the cells, the DATS and DATS-SLNs need cytoplasmic efflux via P-glycoprotein (P-gp) pumps. FA-DATS-SLNs can enter cells via receptor-mediated endocytosis, are unrelated to P-gp efflux, and are located in higher concentrations within TNBS cells. Therefore, functionalization of the DATS–SLNs surface definitely improves the anticancer efficacy of the DATS in TNBC cells.

#### 2.7.7. Apoptotic Protein Bcl2 Inhibition Efficacy

Apoptosis inhibition is one of the hallmarks of cancer. Tumor cell death mechanisms include cell cycle arrest, anti-angiogenesis, anti-*metastasis*, and autophagy, although the great majority of anti-cancer medications have cytotoxic effects on apoptotic signaling pathways in tumors. The apoptosis signaling pathway is regulated by complicated molecular cascade events in the network, which are connected to changes in the expression of certain pro-apoptotic and anti-apoptotic proteins. Pro-apoptotic proteins (Bim, Bax, and Bad) and anti-apoptotic proteins (Bcl2 family proteins) are members of the Bcl2 family (BclxL, Mcl-1, and Bcl2). These proteins are involved in the translocation of mitochondrial mediators as well as in the activation of caspases. Overexpression of the anti-apoptotic protein Bcl2 suppresses apoptosis by blocking mitochondrial outer membrane permeabilization. 

Caspase activation (initiator caspase-9 and effector caspase 3) is associated with the mitochondrial mediator cytochrome-C in the intrinsic route. Pro-apoptotic proteins (Bax, Bad, and Bid) and anti-apoptotic proteins (Bax, Bad, and Bid) regulate cytochrome c migration to the cytosol (Bcl2 and BclxL). Caspase-3, a cysteine protease, is triggered by both death receptors and intracellular/mitochondrial apoptotic signals. Caspase-3 deficiency is associated with BC because it is the primary effector protease that causes cell death by cleaving a wide range of death substrates. When compared with DATS-SLNs and free-DATS, FA-DATS-SLNs had higher levels of Bax, Bad, Caspase-9, and Caspase-3 and lower levels of the anti-apoptotic protein Bcl2 (Figure 5D). This indicates that FA-DATS-SLNs promoted cell death via an intrinsic signaling route. 

A Western blot analysis revealed that FA-DATS-SLNs treatment increased the expression of BAX, BAD, Caspase-3, and -9, and decreased the expression of Bcl2 in MDA-MB-231 TNBC cell lines. Western blot analysis strongly demonstrated the protein expression findings of FA-DATS-SLNs at the molecular level. FA-DATS-SLNs in particular demonstrated increased effectiveness through site-specific drug delivery. However, the elevated activity of DATS, which encapsulated SLNs and functionalized them with FA induced a greater apoptotic response. To treat TNBC cells, current chemotherapeutics in clinical and pre-clinical studies require target-specific administration, which can lessen numerous toxicities and side effects. Overall, FA functionalization on the surface of DATS-SLNs outperformed DATS-SLNs and DATS in terms of targeted and localized action.

## 3. Materials and Methods

### 3.1. Materials

Active Pharmaceutics Ingredient: Diallyl Trisulfide (DATS) was procured from Santa Cruz Biotechnology, India.

SLNs materials: SLNs formulation lipids: palmitic acid, stearic acid, glyceryl monostearate, glyceryl monooleate, cetyl palmitate, and cholesterol; surfactant: Pluronic F-68, Pluronic F-127, Pluronic P-85, Tween 20, Tween 60, and Tween 80; and co-surfactant: soy lecithin, purchased from SD-Fine Chemicals Limited, Mumbai, India. 

Functionalized materials: Folic acid (FA) procured from Sigma-Aldrich, Missouri, USA. All the chemicals are used as procured, without any purification or alteration.

Cell Lines: MCF-7 (non-TNBC), MDA-MB-231 (TNBC), and MCF-10A (non-malignant) procured from India’s National Center for Cell Science, Pune, India.

### 3.2. Formulation and Evaluation of FA-DATS-SLNs 

#### 3.2.1. Lipids Selection for FA-DATS-SLNs Formulation

The solubilizing capacity of DATS was estimated by dissolving an extra amount of the drug in 200 mg of lipids (palmitic acid, stearic acid, glyceryl monostearate, glyceryl monooleate, cetyl palmitate, and cholesterol) in a 5 mL stoppered vial, and then the mixture was mixed by a vortex mixer. The sample vials were then kept at 37 ± 2 °C in an isothermal shaker (REMI, Mumbai, India) for 72 h to achieve equilibrium [31]. The dose was increased by 10 mg intervals. The amount of lipids was chosen as the endpoint when no more DATS solubilization was achievable. The equilibrated samples were transferred to a centrifuge tube and rotated at 3500 rpm for 20 min. The supernatant, obtained from the centrifuge, was filtered through a 0.45 μm filter membrane. The amount of DATS was determined by a UV spectrophotometer at 240 nm for each lipid.

#### 3.2.2. Surfactant Selection for FA-DATS-SLNs Formulation

Nanoparticles were formulated with the specified lipid and analyzed for PS, PDI, and entrapment efficiency using various surfactants (such as Pluronic F-68, Pluronic F-127, Pluronic P-85, Tween 20, Tween 60, and Tween 80). The surfactants were chosen based on their PS and PDI [32].

#### 3.2.3. DATS–Lipid–Surfactant Compatibility Analysis 

DATS, solid lipid, and DATS-SLNs FT-IR spectra (area 4000–500 cm^−1^) were studied using an FT-IR spectrophotometer (Bruker Alpha-One, Bruker Optik, Ettlingen Germany) [33].

#### 3.2.4. DoE Approach for Optimization of DATS-SLNs 

The preliminary screening step in the Design of Experiment (DoE) (V. 7.0, StatEase Inc., Minneapolis, MN, USA) is the most important for choosing crucial formulation parameters and manufacturing qualities that affect critical quality attributes. DATS-SLNs were screened to achieve a minimum PS ≤ 200 nm (particle size influences circulation time, particularly in narrow capillaries where blockage is possible), a narrower PDI value of ≤0.2, and a maximum entrapment efficiency (%EE), (%EE represents the estimated percentage of DATS as the difference between the initial DATS quantity and the unentrapped quantity of DATS in the supernatant with respect to the total amount incorporated in SLNs). Based on the drug’s solubilizing capacity in the lipid, the drug-to-lipid ratio and lipids were assessed for optimization [16,33].

- Optimization of DATS-SLNs using Box–Behnken Design (BBD)

Finally, based on early screening data, a three-level, 3^3^ design (BBD) was selected for DATS-SLNs optimization [16,33]. The optimization was carried out by analyzing the relationship between dependent variables such as PS, PDI, and %EE and critical manufacturing attributes such as the quantity of palmitic acid, amount of surfactant Pluronic F-68, and concentration of co-surfactant soy lecithin, which were optimized at three levels: high, medium, and low (Table 2). The variables were optimized using design expert software (Version 11, Stat-Ease Inc., Minneapolis, MN, USA). A total of 17 runs were screened based on the design in order to find the optimized formulation, and the results were documented. Additionally, the DoE generated a polynomial equation to comprehend how factors affected results. In order to compare various statistical parameters, such as the coefficient of variation (CV), multiple correlation coefficient (R^2^), adjusted multiple correlation coefficient (adjusted R^2^), predicted residual sum of squares, and graphically by a 3D response surface plot, it was statistically determined which experimental model (linear, two-factor interaction, quadratic, and cubic) fit the data the best. The program suggested the model with the best significance value and highest determination coefficients for the chosen probability. The generated mathematical polynomial equations were then statistically validated using the software’s ANOVA (V. 7.0, StatEase Inc., Minneapolis, MN, USA) feature. A *p*-value of 0.05 or less was considered statistically significant.

- Preparation of DATS-SLN

DATS-SLNs were developed using the hot homogenization process [34]. DATS was dissolved in molten palmitic acid lipid. The hot lipid mixture was dispersed in the hot aqueous phase of surfactant Pluronic F-68 and co-surfactant soy lecithin solution and continuously stirred to produce a coarse o/w emulsion. A high-pressure homogenizer was used to homogenize it at a temperature beyond the lipid’s melting point (around 70 °C), producing an oil–water nano-emulsion that was subsequently cooled to room temperature to solidify and create solid lipid nanoparticles. The final formulation was lyophilized and stored for further use.

#### 3.2.5. Functionalization of Folic Acid to Formulate FA-DATS-SLNs 

The surfactant Pluronic F68 was functionalized with FA because it forms the outermost layer of the SLNs to create a protective layer for the drug-loaded core. For the FA–Pluronic F68 formulation, 10 mg of FA was thoroughly mixed in 6 mL of dry dimethyl sulfoxide (DMSO). To activate the FA, 20 mg of 1,1-carbonyldiimidazole (CDI) was added to the solution and mixed in the dark overnight [35]. For the synthesis of the FA–Pluronic F68, the FA-activated solution was mixed with 50 mg of dry Pluronic F68 and agitated at room temperature for another 24 h. The purification of the FA–Pluronic F68 was performed by dialyzing (tube: MWCO 3500) the mixture against deionized water for 1 day. The FA–Pluronic F68 was lyophilized and stored at 4 °C. FA-DATS-SLNs were formulated using the same hot homogenization process as DATS-SLNs but using FA–Pluronic F68. The functionalization was confirmed by FT-IR spectroscopy and NMR analysis.

#### 3.2.6. Evaluation of FA-DATS-SLNs -Entrapment Efficiency, Particle Size, Morphology and *In Vitro* Release Profile 

The entrapment effectiveness of DATS in the lyophilized SLNs formulation was evaluated using UFLC (SPD-M20A, Shimadzu, Kyoto, Japan) at a λ max of 240 nm. DTAS-SLNs were dissolved in 10 mL of a 1:1 mixture of methanol and sodium dodecyl sulfate during the experiment. The solution was centrifuged for 10 min at 32 °C in a super filter tube using a Sigma-3K30 centrifuge (Sigma-Aldrich, Seelze, Germany). Methanol was used to extract the ultra-filtrate, which was then filtered through a 0.45 µm filter. UFLC [36] was used to analyze the supernatant. The mobile phase was acetonitrile and water (75:25, *v*/*v*) with a flow rate of 1.1 mL/min, while the solid phase was a C18 (250 mm 4.6 mm) column. The retention time of the DATS was observed at 9.3 (Figure S5).

The key function of the SLNs was to increase the DATS therapeutic efficacy and stability while ensuring a minimal PS and a high EE. The Malvern Zeta sizer (Nano ZS, Malvern Instruments, Worcestershire, UK) was used [33] to determine the PS (nm) and ZP (mV) of DATS-SLNs and FA-DATS-SLNs at 25 °C.

SEM images (JEOL JSM 7610F, Tokyo, Japan) were used to investigate the morphology of functionalized FA-DATS-SLNs [33,37]. To make the lipid formulation conductive, it was coated for 3 min with palladium gold (auto fine coater JFC-1600). The picture was captured using a field emission SEM based on the JEOL JSM 7610F (Tokyo, Japan).

The dialysis method was used to evaluate DATS release from the FA-DATS-SLNs formulation at two distinct pH values (5.5 and 7.4) at 37 °C. A total of 1 mg of the lyophilized FA-DATS-SLNs were blended in 1 mL of PBS and put in a dialysis tube (MWCO 8 kDa) (HiMedia, Mumbai, India). The samples were immersed in 3 mL of PBS with agitation at 100 rpm at 37 °C. The samples were collected at 1, 4, 8, 12, 24, 48, and 72 h intervals while the sink condition was maintained. DATS release was quantified using UFLC at 240 nm. The experiments were carried out in triplicate, and the data were analyzed graphically [33].

#### 3.2.7. *In Vitro* Cell Line Study for Triple Negative Cancer Efficacy

- *In vitro* cytotoxicity study for evaluation of functionalization

The carcinoma cell lines MCF-7 (human breast adenocarcinoma cell line with estrogen, progesterone, and glucocorticoid receptors present), MDA-MB-231 (highly aggressive triple-negative breast cancer (TNBC) cell line that lacks estrogen, progesterone, and human epidermal growth factor receptor), and non-carcinogenic MCF-10A (epithelial cell line isolated from female mammary gland) were used in the FA-DATS-SLNs efficacy experiment. The cytotoxicity of the formulation was evaluated using the 3-(4,5-dimethylthiazol-2-yl)-2,5-diphenyltetrazolium bromide (MTT) assay by the EZ-Cytox Cytotoxicity Assay Kit (DoGen Bio, Seoul, South Korea). The cells were seeded into 96-well plates at a density of 5 × 10^3^ cells/well and incubated overnight in Dulbecco’s Modified Eagle Medium (DMEM). Next, the medium was removed and cells were washed with 1 mL PBS. Then, the cells were treated with different concentrations (0, 0.625, 1.25, 2.5, 5, and 10 µM in serum-free media) of DATS, DATS-SLNs, and FA-DATS-SLNs and incubated for 48 h at 37 °C under 5% CO_2_ conditions. After 48 h, the medium was removed, and the cells were washed with 1 mL of PBS. Following that, 20 µL of MTT solution (stock: 5.0 mg/mL in PBS) from the EZ-Cytox Cytotoxicity Assay Kit [38] was poured into each well. The cells were incubated for 4 h to allow mitochondrial dehydrogenases to activate. Finally, the absorbance of the formazan was measured at 450 nm using a microplate reader (VICTORTM X3, PerkinElmer). The experiment was carried out in triplicate. Untreated control cells exhibited MTT-like cytotoxicity. To compute the concentration of each formulation that reduced growth by 50% and the 95% confidence interval, GraphPad Prism 7.0 (GraphPad Software, Inc., San Diego, CA, USA) software was used to run a nonlinear regression analysis and generate a dose–response curve.

The Selectivity Index (SI) was calculated by dividing the cytotoxicity (IC_50_) into normal cells (MCF-10A) by the cancer cell cytotoxicity (IC_50_) (MCF-7 and MDA-MB 231). Treatments with a SI value greater than 3 were shown to be preferentially cytotoxic to MCF-7 and MDA-MB231 cells.

- Colony formation assay for long term cell cytotoxicity effect of conjugates

The clonogenic assay was performed to determine the long-term impact of a cytotoxic substance for 7 days. In the current investigation, the TNBC cell line MDA-MB-231 was plated in triplicate onto 6-well plates at a density of 2000 cells/well. After 24 h, the cells were washed with PBS. DATS, DATS-SLNs, and FA-DATS-SLNs were added to a 37 °C environment with 95% air and 5% CO_2_ and incubated for 7 days. On the 7th day, the supernatant was removed and washed with PBS. Methanol and formaldehyde (3:1) were used to fix the cells, and the colonies were stained with 0.5% crystal violet for 15 min. The excess color was washed away with distilled water. The colonies with more than 50 cells were counted using a microscope [39]. The number of colonies was used to calculate cell survival following long-term treatment with the compounds.

- Cell migration assay

Cell migration was studied using a scratch assay. MDA-MB-231 (3 × 10^5^) was planted in 6 wells. The scratch was formed by scraping with a microtip, and the plates were washed with serum-free media. The cells were cultured for 24 h with DATS-SLNs and FA-DATS-SLNs. The cells were imaged at 0, 24, 36, and 48 h using an Olympus microscope model IX-81 (Tokyo, Japan).

- DNA fragmentation analysis of functionalization efficacy for apoptosis

DNA fragmentation was used as a qualitative marker for cell apoptosis. The DNA ladder was compared with API-treated and control DMSO-treated MDA-MB-231 cells. In a nutshell, 5 × 10^6^ cells/mL of cancer cells were transported to a lysis buffer at 65 °C [40]. The cells were treated with 7.1 µg/mL concentration of DATS and an equivalent concentration of DATS in DATS-SLNs and FA-DATS-SLNs formulations. The DNA was extracted using phenol/chloroform/isoamyl-alcohol (25:24:1 *v*/*v*), followed by ethanol precipitation after treatment. The DNA was re-suspended in Tris-EDTA buffer, pH 8.0, at 37 °C for 1 h. The extracted DNA was mixed with 1X loading dye and run in triplicate for 15 min on a 2% agarose gel at 50 V. A UV trans-illuminator was used to image the fragmented DNA (Bio-Rad, Hercules, CA, USA).

- TNBC Cellular internalization of functionalized FA-DATS-SLNs

LSCM live cell imaging analyzed the cellular initialization of the FA-DATS-SLNs and DATS-SLNs. In a confocal culture dish, 2 × 10^3^ MDA-MB-231 cells were plated and left for incubation overnight. Cells were stained with Nile Red (0.3 μg/mL) at 37 °C for 30 min. Cells were washed three times with PBS and then again stained with ER-Tracker™ Green and DAPI at 37 °C for 30 min. Cells were monitored through the confocal microscope (LSCM, A1Plus, Nikon, Tokyo, Japan) for 2 h to estimate the cellular uptake of DATS-SLNs and FA-DATS-SLNs [41,42]. The image was analyzed by software (NIS-E, Ver.4.00 Nikon).

- Apoptosis quantification of conjugates efficacy by flow cytometry

MDA-MB-231 cells (1 × 10^5^ cells/well) were plated in 6-well plates for 24 h. Cells were treated with the 7.1 µg/mL concentration of DATS and the equivalent concentration of DATS in DATS-SLNs and FA-DATS-SLNs formulations. Treated cells were trypsinized, collected, and centrifuged at 15,000 rpm for 3 min. The supernatant was removed, and the cells were washed twice with 1 mL of PBS. Cells were stained with 5 µL of annexin V-FITC and 10µL of Propidium iodide (PI) and incubated for 45 min in dark conditions. The % of annexin-V-positive cells was used to calculate the degree of apoptosis. Flow cytometry analysis of Annexin V/PI-stained cells was provided for cell quantification. Annexin V-negative and PI-negative cells indicate living cells; annexin V-positive but PI-negative cells indicate early apoptotic cells; and annexin V-positive and PI-positive cells indicate late apoptotic and necrotic cells [43].

- Apoptotic protein Bcl2 inhibition efficacy

MAD-MB-231 cells (1 × 10^5^ cells/well) were grown in 6-well plates and treated with DATS, DATS-SLNs, and FA-DATS-SLNs for 24 h. Following treatment, the cells were lysed using a radio-immunoprecipitation assay buffer and centrifuged (12,000× *g*, 30 min) at 4 °C. The total protein content of the supernatant was measured using the Bradford technique [44,45]. In 10% SDS-PAGE, proteins (30 g/lane) were electrophoresed. Following that, PVDF membranes were incubated overnight at 4 °C with primary antibodies Caspase-3 (1:500) and Caspase-9 (1:1000), Bax (1:200), Bad (1:1000), and Bcl2 (1:200). GAPDH was utilized as a control. Following incubation, the membrane was washed twice with TBST buffer. The membrane was then treated for 1 h with the secondary antibody, goat anti-rabbit IgG coupled with horseradish peroxidase. The band intensities of the DATS, DATS-SLNs, and FA-DATS-SLNs were compared.

## 4. Conclusions

FA-DATS-SLNs formulated with surface-functionalized FA outperformed non-targeted DATS and DATS-SLNs in terms of cellular absorption, resulting in increased cytotoxicity. FA functionalization boosted target selectivity toward aggressive TNBC MDA-MB-231 cells and has proven to be a successful TNBC therapeutic. FA functionalization successfully overcomes DATS-SLNs’ off-targeting limitation. FA-DATS-SLNs were more cytotoxic than DATS and DATS-SLNs, perhaps due to FA ligands’ potential to disrupt intrinsic apoptotic signaling pathways. FA-DATS-SLNs significantly downregulate anti-apoptotic proteins (Bcl2) while upregulating pro-apoptotic caspase-9 and enhancing the apoptotic potential of the functionalized formulation by interfering with the intrinsic apoptotic pathway. Thus, the targeting ligand (FA) improved the effectiveness of FA-DATS-SLNs, and this study might open the door for targeted therapy of DATS for the treatment of TNBC using a safe drug delivery vehicle with minimal side effects. However, it should be noted that this is primarily a proof-of-concept study for the construction of FA-functionalized DATS-SLNs for TNBC management. Further in vivo research is needed to assess treatment effectiveness.

## Figures and Tables

**Table 1:** IC50 value and Selectivity Index of DATS, DATS-SLNs, and FA-DATS-SLNs on MCF-7, MDA-MB-231, and MCF-10A cell lines.

Cell Line	IC_50_ (µg/mL) Value at 48 h		
	DATS	DATS-SLNs	FA-DATS-SLNs
MCF-7	>48.8	>46.2	10.8
MDA-MB-231	>55.5	>50.5	7.1
MCF-10A	>96.7	>98.6	93.3
SI for MCF-10A/MCF-7	1.98	2.4	8.7
SI for MCF-10A/MDA-MB-231	1.75	2.1	13.2

**Table 2:** Dependent and Independent variables for BBD model of DATS-SLNs.

Levels			
Factors	Low	Medium	High
**Independent variables**			
Lipid: Palmitic acid (mg) (X1)	50	75	100
Surfactant: Pluronic F-68 (%)(X2)	1.5	2	2.5
Co surfactant: Soy lecithin (%)(X3)	0.25	0.5	0.75
**Dependent variables**			
PS (nm) (Y1)	Minimize		
EE (%) (Y2)	Maximize		

## Data Availability

Not applicable.

## Supplementary Materials

The following supporting information can be downloaded at: https://www.mdpi.com/article/10.3390/molecules28031393/s1, Figure S1: Compatibility study of the lipid palmitic acid with drug DATS in physical mixture as well as in DATS-SLNs formulation; Table S1: BBD model for the DATS-SLNs dependent and independent parameters; Table S2: Analysis of variance (ANOVA) for response surface quadratic model for DATS-SLNs PS; Table S3: Analysis of variance (ANOVA) for response surface quadratic model for DATS-SLNs %EE; Figure S2: Particle size distribution of DATS-SLNs; Figure S3: FTIR conformation of the FA functionalization on the surface of DATS-SLNs; Figure S4: Particle size distribution of FA-DATS- SLNs. Figure S5: UFLC chromatogram of DATS using mobile phase of acetonitrile and water (75:25, *v*/*v*), flow rate 1.1 mL/mim, C18 (250 mm 4.6 mm) column. Retention time of the DATS was observed at 9.3.

## Sample Availability

Samples of the compounds are available from the authors.
