# Supplementary material for: Folic Acid Functionalized Diallyl Trisulfide–Solid Lipid Nanoparticles for Targeting Triple Negative Breast Cancer
Source: Molecules. 2023 Feb 1;28(3):1393. doi: 10.3390/molecules28031393 (PMC9921651; doi:10.3390/molecules28031393)
Supplement: Supplementary file 1 [file molecules-28-01393-s001.zip › molecules-2178334-supplementary.pdf]

Supplementary File

Folic Acid Functionalized Diallyl Trisulfide–Solid Lipid Nanoparticles for Targeting Triple Negative Breast Cancer

Anindita De [1,*], Parikshit Roychowdhury [2], Nihar Ranjan Bhuyan [2], Young Tag Ko [1], Sachin Kumar Singh [3], Kamal Dua [4,5] and Gowthamarajan Kuppusamy [6,*]

[1] College of Pharmacy, Gachon Institute of Pharmaceutical Science, Gachon University, Incheon 21936, Republic of Korea
[2] Department of Pharmaceutical Chemistry, Himalayan Pharmacy Institute, Majitar 737136, East Sikkim, India
[3] School of Pharmaceutical Sciences, Lovely Professional University, Phagwara 144411, Punjab, India
[4] Faculty of Health, Australian Research Centre in Complementary & Integrative Medicine, University of Technology Sydney, Ultimo, NSW 2007, Australia
[5] Discipline of Pharmacy, Graduate School of Health, University of Technology Sydney, Ultimo, NSW2007, Australia
[6] Department of Pharmaceutics, JSS College of Pharmacy, JSS Academy of Higher Education & Research, Ooty, Nilgiris 643001, Tamil Nadu, India
* Correspondence: aninditanirupa@gmail.com (A.D.); gowthamsang@jssuni.edu.in (G.K.); Tel.: +82-1098218750 (A.D.); +91-9443089812 (G.K.)

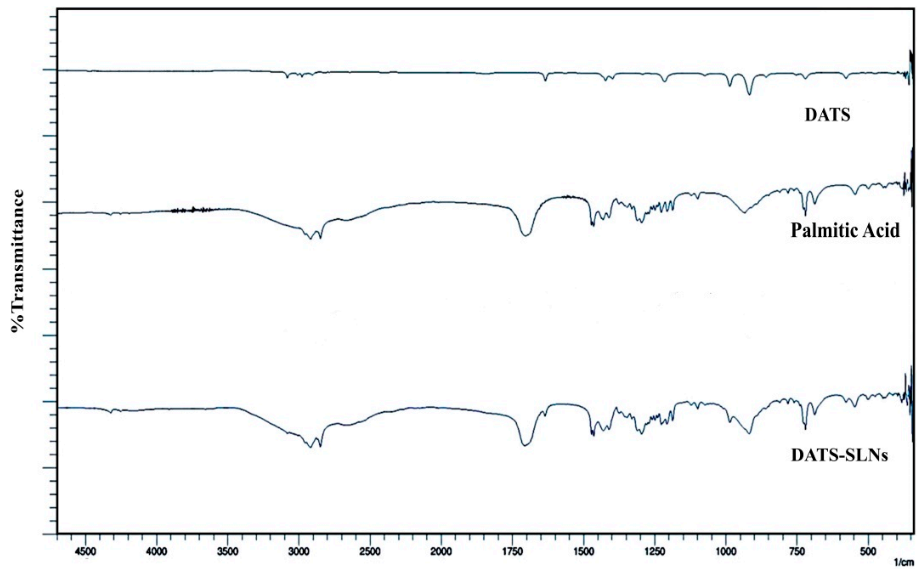

Figure S1. Compatibility study of the lipid Palmitic acid with drug DATS in DATS-SLNs formulation.

Table S1. BBD model for the DATS-SLNs dependent and independent parameters.

Std. Order	Run	Block	Factor 1 A: Lipid (mg)	Factor 2 B: Surfactant (%)	Factor 3 C: Co.surfactant (%)	Response 1: PS (nm)	Response 2: EE (%)
9	1	1	75	1.5	0.25	216.25	40.27
4	2	1	100	2.5	0.5	148.3	55.89
15	3	1	75	2	0.5	174.1	57.32
11	4	1	75	1.5	0.75	149.5	60.18
17	5	1	75	2	0.5	166.4	63.12
1	6	1	50	1.5	0.5	106.29	73.2
6	7	1	100	2	0.25	188.12	59.52

5	8	1	50	2	0.25	140.12	58.7
12	9	1	75	2.5	0.75	192.36	47.4
3	10	1	50	1.5	0.5	106.29	73.2
2	11	1	100	1.5	0.5	202.62	50.71
10	12	1	75	2.5	0.25	117.51	46.34
13	13	1	75	2	0.5	167.23	54.36
16	14	1	75	2	0.5	169.41	60.79
7	15	1	50	1.5	0.5	106.29	73.2
8	16	1	100	2	0.75	214.85	67
14	17	1	75	2	0.5	166.4	60.79

Table S2. Analysis of variance (ANOVA) for response surface quadratic model for DATS-SLNs PS.

	**F value**	***p* Value Prob > F**	**Remarks**
Quadratic model			Significant ($p < .05$)
A-Lipid	326.1461617	<.0001	
B-Surfactant	1800.434914	<.0001	
C-Co surfactant	198.3049652	<.0001	
AB	1.995245264	0.1326	
AC	79.17655501	<.0001	
BC	102.6474495	<.0001	
A^2	749.8825748	<.0001	
B^2	166.0984932	<.0001	
C^2	115.785255	<.0001	
Residual	111.0032407	<.0001	
Lack of fit	0.132330929	0.8424	Not significant

Table S3. Analysis of variance (ANOVA) for response surface quadratic model for DATS-SLNs %EE.

	**F value**	***p* Value Prob > F**	
Quadratic model	17.51924	0.0008	Significant ($p < .05$)
A-Lipid	13.88987	0.009	
B-Surfactant	1.999535	0.1463	
C-Co surfactant	32.18754	0.0006	
AB	8.798451	0.022	
AC	5.124361	0.062	
BC	12.47919	0.0143	
A^2	35.21838	0.0008	
B^2	46.61263	0.0003	
C^2	0.817536	0.0078	
Residual			
Lack of fit	0.34651	0.7101	Not significant

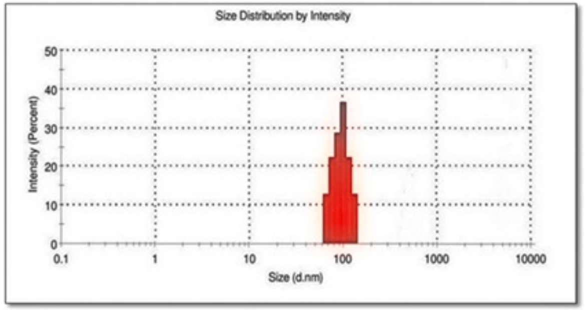

Figure S2. Particle size distribution of DATS-SLNs.

(A)

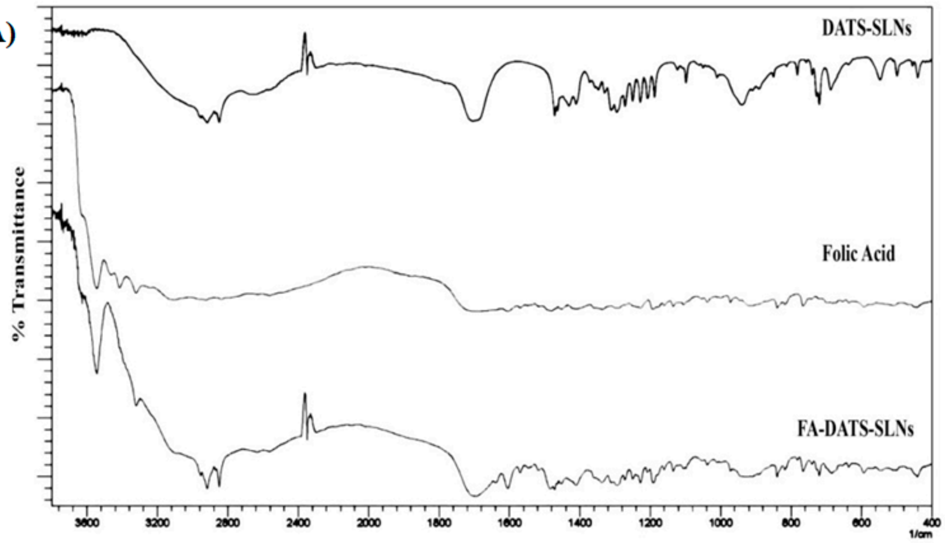
DATS-SLNs
Folic Acid
FA-DATS-SLNs
% Transmittance
3600
3200
2800
2400
2000
1600
1400
1200
1000
800
600
400
1/cm

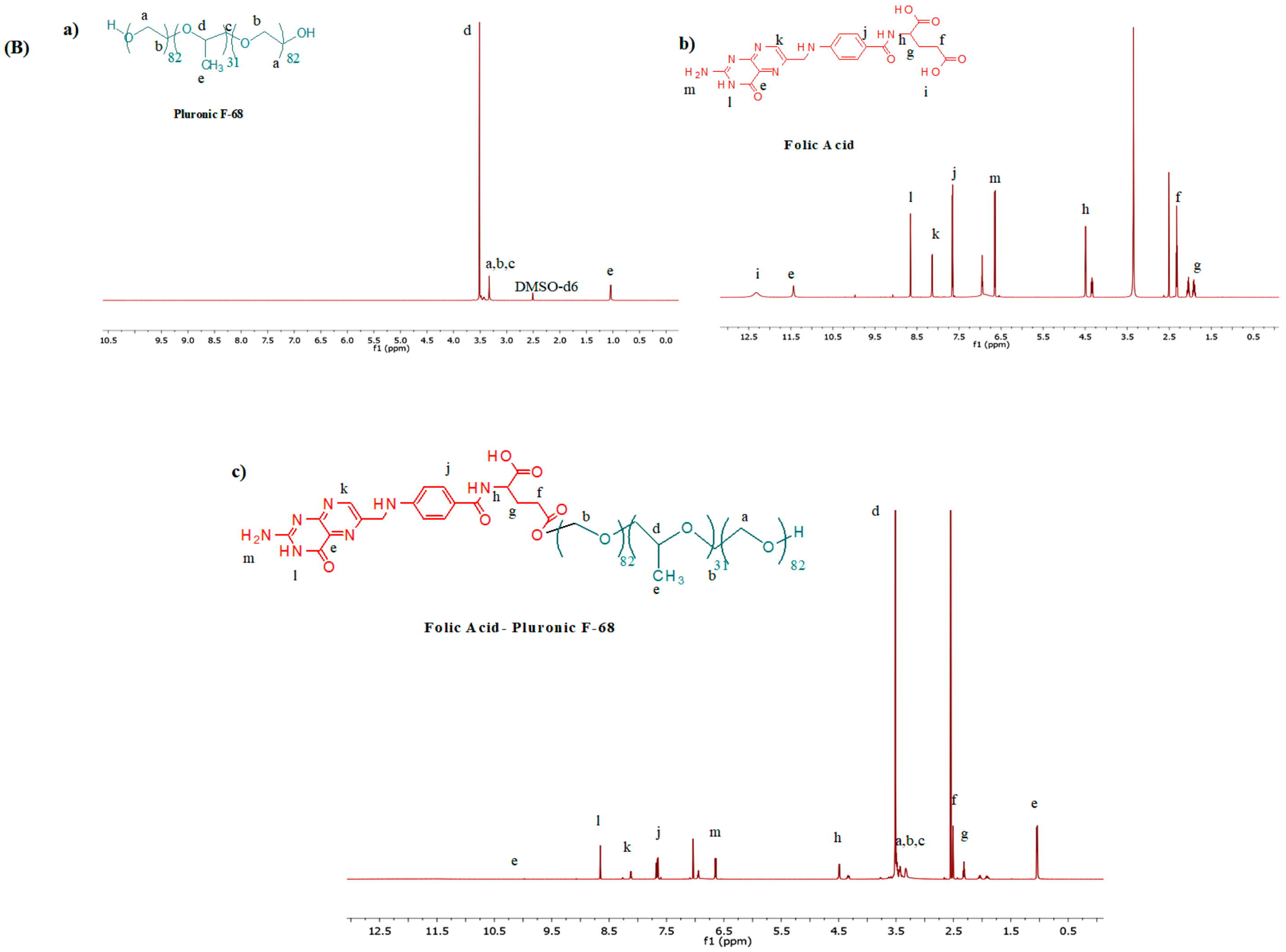

Figure S3. A: FTIR conformation of the FA functionalization on the surface of DATS-SLNs. **B.** The NMR spectrum of Pluronic F68, Folic acid and FA-Pluronic F68 for conjugation conformation.

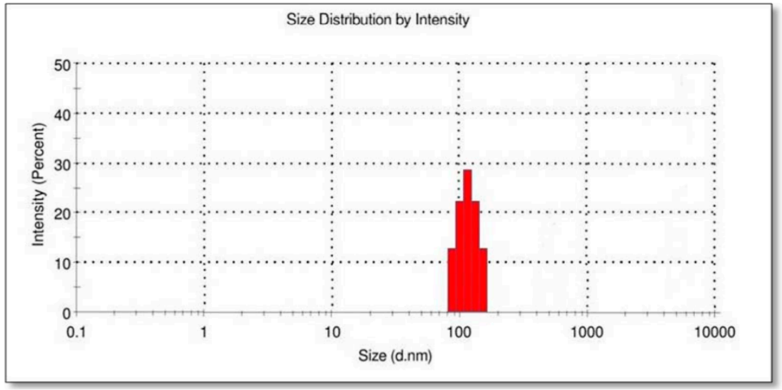

Figure S4. Particle size distribution of FA-DATS- SLNs .

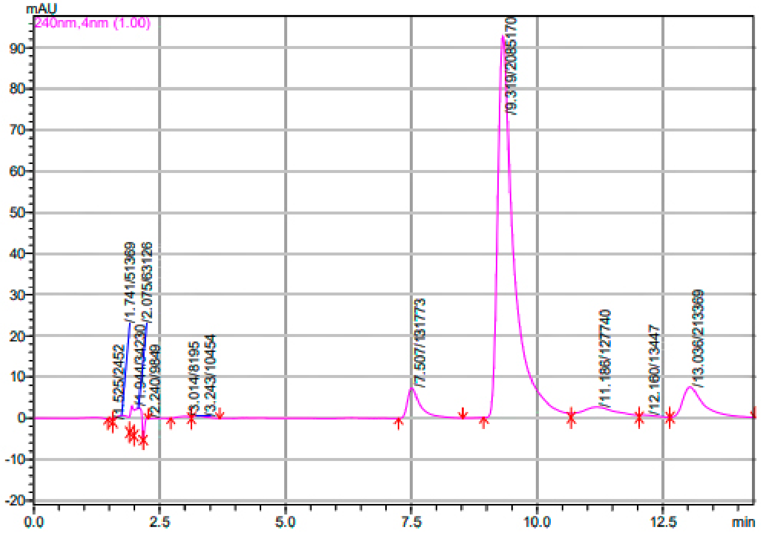

Figure S5. UFLC chromatogram of DATS using mobile phase of acetonitrile and water (75:25, v/v), flow rate of 1.1 ml/min, C18 (250 mm 4.6 mm) column. The retention time of the DATS was observed at 9.3.
